# Development of an early-warning system for high-risk patients for suicide attempt using deep learning and electronic health records

**DOI:** 10.1038/s41398-020-0684-2

**Published:** 2020-02-20

**Authors:** Le Zheng, Oliver Wang, Shiying Hao, Chengyin Ye, Modi Liu, Minjie Xia, Alex N. Sabo, Liliana Markovic, Frank Stearns, Laura Kanov, Karl G. Sylvester, Eric Widen, Doff B. McElhinney, Wei Zhang, Jiayu Liao, Xuefeng B. Ling

**Affiliations:** 1 Department of Cardiothoracic Surgery, Stanford University, Stanford, CA USA; 2 Clinical and Translational Research Program, Betty Irene Moore Children’s Heart Center, Lucile Packard Children’s Hospital, Palo Alto, CA USA; 3 HBI Solutions Inc, Palo Alto, CA USA; 4 Department of Health Management, Hangzhou Normal University, Hangzhou, China; 5 Department of Psychiatry and Behavioral Sciences, Berkshire Medical Center, Pittsfield, MA USA; 6 Department of Psychiatry and Behavioral Sciences, University of Massachusetts Medical School, Worcester, MA USA; 7 Department of Surgery, Stanford University, Stanford, CA USA; 8 Department of Psychiatry, West China Hospital of Sichuan University, Chengdu, China; 9 Department of Bioengineering, Bourns College of Engineering, University of California at Riverside, Riverside, CA USA; 10 West China-California Center for Predictive Intervention Medicine, West China Hospital, Sichuan University, Chengdu, Sichuan China

**Keywords:** Psychiatric disorders, Scientific community

## Abstract

Suicide is the tenth leading cause of death in the United States (US). An early-warning system (EWS) for suicide attempt could prove valuable for identifying those at risk of suicide attempts, and analyzing the contribution of repeated attempts to the risk of eventual death by suicide. In this study we sought to develop an EWS for high-risk suicide attempt patients through the development of a population-based risk stratification surveillance system. Advanced machine-learning algorithms and deep neural networks were utilized to build models with the data from electronic health records (EHRs). A final risk score was calculated for each individual and calibrated to indicate the probability of a suicide attempt in the following 1-year time period. Risk scores were subjected to individual-level analysis in order to aid in the interpretation of the results for health-care providers managing the at-risk cohorts. The 1-year suicide attempt risk model attained an area under the curve (AUC ROC) of 0.792 and 0.769 in the retrospective and prospective cohorts, respectively. The suicide attempt rate in the “very high risk” category was 60 times greater than the population baseline when tested in the prospective cohorts. Mental health disorders including depression, bipolar disorders and anxiety, along with substance abuse, impulse control disorders, clinical utilization indicators, and socioeconomic determinants were recognized as significant features associated with incident suicide attempt.

## Introduction

Suicide is the tenth leading cause of death in the US, claiming the lives of more than 44,000 individuals in 2015. Over the past 15 years, the suicide rate has increased 24% from 10.5 (in 1999) to 13.7 (in 2015) per 100,000 people^1^. Suicide is the third leading cause of death among individuals between the ages of 10 and 14, and the second leading cause of death among individuals between the ages of 15 and 34 ^2^. Rates of suicide in several specific demographics, including veterans and native Americans, consistently exceed the national average^2,3^. According to a CDC report, suicide accounted for economic losses of $50.8 billion in 2013, representing 24% of fatal injury costs^2^. A suicide attempt is a nonfatal, self-directed, potentially injurious behavior with lethal intent. Data suggest that approximately 25 people harm themselves for every reported death by suicide^4^. Many suicide attempts, however, go unreported or untreated^4^. Surveys suggest that over one million people in the US each year engage in intentionally inflicted self-harm, and 0.6% of adults age 18 and older in the United States attempted suicide in 2015 ^5^. Since the presence of previous suicide attempts is the most powerful predictor of eventual death by suicide, efficiently identifying prior suicide attempts is a critical step towards reducing suicide deaths and saving lives^6^.

Various cohorts have shown that somewhere between 56 and 68% of suicides die on the first attempt, the index attempt^7–11^. Of the 32–44% who survive the index attempt and receive emergency or hospital level of care, rates of subsequent completed suicide are exceptionally high, ranging from 2.3 to 4%^9,12–15^. Thus, a previous suicide attempt confers a very high risk of subsequent death by suicide. In one study^9^, 82% of the subsequent suicides in these hospitalized or ED-treated suicide attempt survivors occurred within 1 year of the index attempt. Evidence from clinical trials and research suggests that encouraging help-seeking behaviors and increasing the likelihood of intervention by a third party are valuable strategies to reduce suicide in hotspots^16^. Interventions for those high risk of suicide attempts are extremely important, and may help in preventing death by suicide. Therefore, suicide attempt prediction tool to stratify individuals into different risk groups at the population level would be useful in that it can assist providers in reaching the most vulnerable.

Various efforts have been made to identify risk factors of suicide thoughts and behaviors, and to predict the probability of future suicide attempts. Although a few high-performance models were reported in studies where the cohorts were enriched for cases^17–21^, prediction accuracy was limited when applied to a general population where the incidence of suicide attempts was extremely low^22^. While univariate and multivariate analyses have been successful in revealing the different roles of individual-level and population-level factors in suicide attempts, reasons for a suicide attempt could be complex and associated to a multi-level network^23^. Moreover, although some risk factors have higher weights than others in a specific model of predicting suicide attempt, the meta-analysis found that there is not a dominant factor that has significantly larger importance than the rest. According to a meta-analysis that summarized studies on suicide risk factors over the past 50 years^24^, there are two future directions: (1) the implementation of advanced machine-learning technology to incorporate the relations between different risk factors; (2) the utilization of a high-dimensional dataset containing comprehensive clinical profile of patients. The two directions are supportive to each other, in that the advanced machine-learning techniques can make the best of a large number of features through constructing a complex network to approach the outcome, and a large, high-dimension dataset ensure an effective use of the learning techniques and maximizes the power of the algorithm.

Therefore, using a large, longitudinal electronic health records (EHRs) routinely captured by hospitals, we applied deep learning methodology to develop a neural network model, and validated it within a different population. Our study focused on a short-term prediction (within 1 year) to support the clinical utility in decision making^25^, and extended the concepts of early-warning system (EWS) to the next 1-year suicide attempt surveillance of a general population.

## Methods

### Ethics statement

Protected personal health information was removed for the purpose of this research. Since the present study was conducted using deidentified data, this study was also exempted from ethics review by the Stanford University Institutional Review Board (September 26, 2018).

### The suicide attempt early-warning system (EWS)

**Fig. 1:**
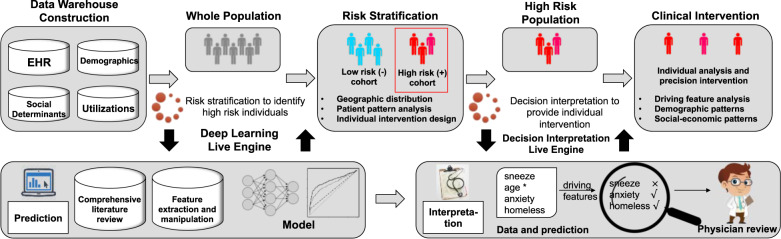
Development of risk of suicide attempt early-warning system. The system is consist of the deep learning live engine and the decision interpretation live engine. The deep learning engine is design to provide a real-time risk stratification for the whole population, so that the high-risk population can be found in advance. The decision interpretation live engine is used to analyze the driving features of the high-risk population and help provide insight for individual intervention.

Early-warning systems are tools used by health-care providers to recognize the early signs of a serious and potentially life-threatening clinical deterioration in order to initiate mitigating interventions and management^26–30^. The key to a successful EWS is to accurately identify high-risk patients for which mitigating interventions exist or can be developed with a high degree of efficacy. Therefore, the core functions of the suicide attempt EWS are to stratify a defined population into different risk subcohorts according to probability of suicide attempt and align high-risk cohorts according to threshold triggers with timely interventions determined by a mental health expert. It mainly consists of three steps, i.e., data warehouse construction, risk stratification, and clinical intervention (Fig. 1).

### Berkshire Health System dataset

**Fig. 2:**
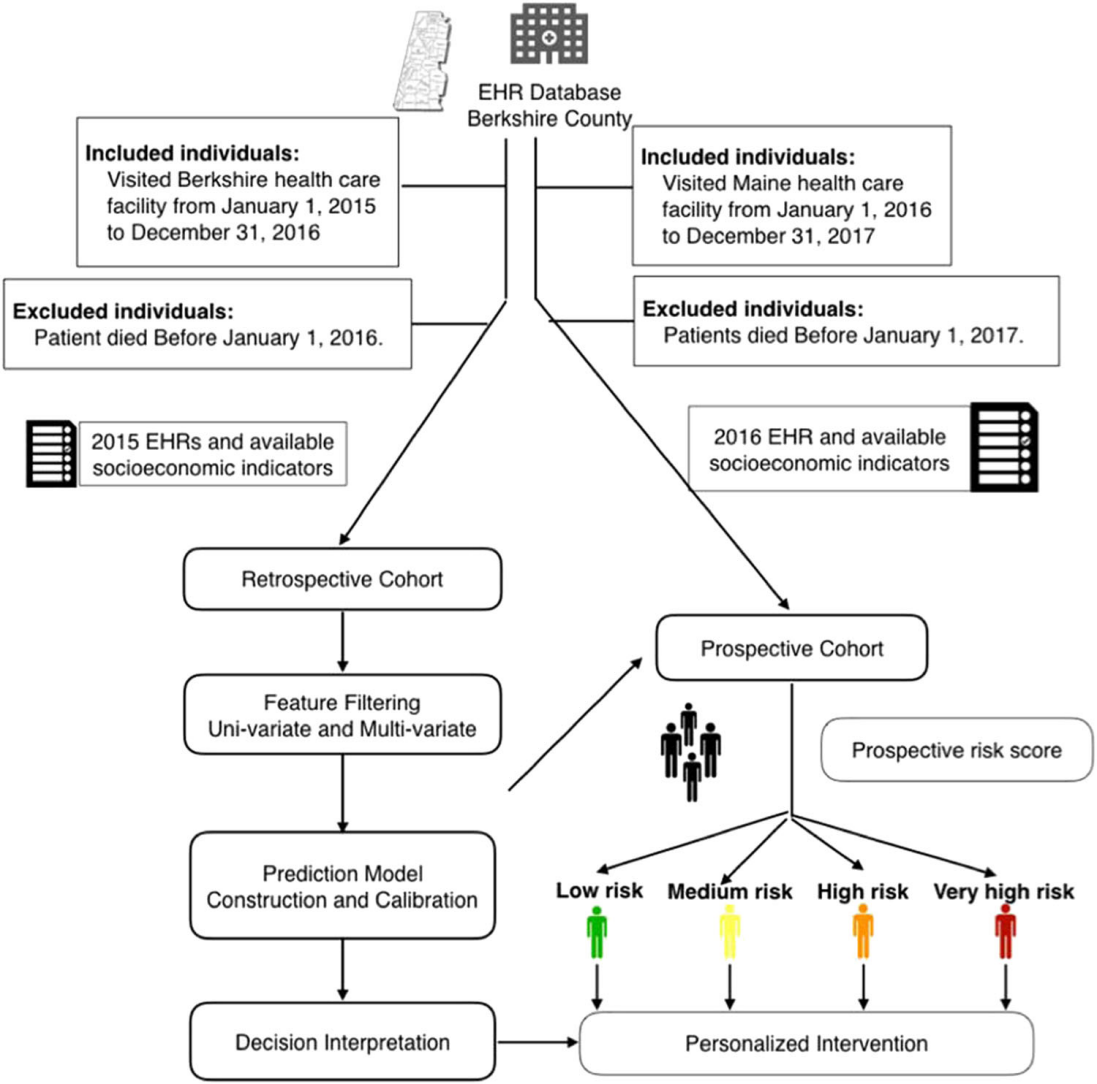
Workflow diagram depicting model construction and evaluation. The retrospective cohort consisted of 118,252 individuals with EHR profiles extracted from 2015, 255 of whom (cases) attempted suicide in 2016. The validation cohort consisted of 118,095 individuals, with EHR profiles extracted from 2014, 203 of who were admitted for suicide attempt in 2017.

The analyzed dataset was derived from the EHR of all patients that visited any of the three Berkshire Health System hospitals from January 1, 2015 to December 31, 2017. The detailed inclusion and exclusion criteria are demonstrated in the study design workflow (Fig. 2).

#### Definition of suicide attempt

Suicide attempt in this study was defined by the ICD-10-CM diagnosis codes X71 to X83 and T14.91 that refer specifically to suicide and self-inflicted injury. For the prediction modeling cohort, cases in the retrospective population referred to patients who received a new suicide attempt diagnosis during the calendar year of 2016 (from January 1, 2016 to December 31, 2016), while cases in the prospective cohort were patients receiving a suicide attempt diagnosis during the 2017 calendar year (from January 1, 2017 to December 31, 2017).

#### Cohorts

Given that the prediction model was constructed to predict the risk of an individual attempting suicide during the following calendar year based on the medical records from the current year, features from the retrospective cohort were extracted from the clinical and health historical record of 2015, a total of 118,252 patients, 245 of whom attempted suicide in the year 2016. Similarly, the prospective cohort included 118,095 patients and 203 cases with a suicide attempt in the year 2017. The large population size can guarantee the statistical power of the study. ICD-10 was implemented in October 2015 and some of the limitations of data derived during this transition from ICD-9 (including facility-to-facility variation in coding data and clinician-to-clinician variation in recording data and the difference in self-harm with intent to die and self-harm without intent to die) are discussed in a recent publication^31^.

The data were highly imbalanced (with a 0.21% incidence rate), a problem that was attacked on two fronts. One way was to train the model in a case-enriched subcohort. The numbers of cases and the incidence rates of different subcohorts were computed (Supplementary 1). The mental illness subcohort (with 21,013 patients) had more cases (133 out of 245) than the other subcohorts and had nearly three times the incidence rate (0.61% vs. 0.21%) of the total population. Therefore, we chose to train the risk stratification model with the mental illness retrospective subcohort. The other component was to use bootstrapping in the subcohort, to further increase the incidence rate to 5%. The demographic baseline was shown in Supplementary 2.

### Prediction model construction and evaluation

#### Features

All the clinical history profiles during the preceding 12-month observation window were recorded and processed. Various categories of data were extracted from the original health records, including demographics, essential and secondary diagnoses and procedures using the ICD-10-CM coding system, outpatient medication prescriptions using the RxNorm prescription coding system, and clinical utility records including the counts of emergency visits, inpatient visits, and chronic diseases in the observation window. In order to analyze the community factors associated with suicide attempts, we also considered a number of accessible socioeconomic variables extracted from the US census. Details of coding methods have been detailed previously^32^. The missing data handling method is provided in Supplementary 3. Overall, more than 15,000 features were recruited into the original data pool.

#### Model construction and interpretation

The mental illness retrospective subcohort was utilized to construct the risk stratification model. This process was accomplished in four phases.

##### Feature filtering

The feature filtering phase contains two steps, i.e. the univariate filtering and the multivariate filtering. In the univariate filtering step, the two-sided *t* test was first used to prefilter the features *p* value, and those with *p* value < 0.05 were kept (*N* = 2186). Then an age−gender-adjusted logistic regression was used to compute the odds ratio of each feature. Features with odds ratio > 1.5 or <1/1.5 were kept (*N* = 484). In the multivariate filtering step, the XGBoost algorithm^33^ was utilized to build a multivariate model, and resulted in the final set of 117 features recruited to build the model.

##### Model training

A deep neural network (DNN) comprising an input layer (of 117 dimensions), 3 hidden layers (each 512 dimensions with a “tanh” activation function) and a scalar output layer (one dimension with a “sigmoid” activation function) was trained. The hyper-parameters of the neural network were selected by grid search using the Python library scikit-learn. The tuned hyper-parameters included the network depth, number of hidden units, learning rate, dropout weights, etc. The weighted cross entropy loss function was employed to tune the parameters of the DNN, aiming to penalize the error of misclassifying a case to the control group. In order to demonstrate the effectiveness and accuracy of the DNN model, a multivariate logistic regression model with L-1 regularization and an XGBoost model is trained as baseline models. The input to the two baseline models is identical to that of the DNN model, and the parameters are selected via cross validation.

##### Risk calibration

The DNN estimations $\hat y$ were further mapped to positive predictive values (PPVs)^34^, which could also be interpreted as risk scores that measured probability of suicide attempts within the next year among individuals having predictive estimates identical as or larger than $\hat y$.

##### Decision interpretation

For a very complex model like the DNN, it is important to provide quantitative relationships between the patients’ clinical profiles with the model decisions. In our work, the Local Interpretable Model-agnostic Explanations (LIME)^35^ algorithm was utilized to interpret the risk stratification results. For each patient, let **x** represented the features of the patient. We sampled *M* instances $\left( {{\mathbf{x}}^{(1)},{\mathbf{x}}^{(2)}, \cdots ,{\mathbf{x}}^{(M)}} \right)$ around **x** by drawing nonzero elements of **x** uniformly at random. We labeled these sampled instances with the trained DNN. We optimized (1) to get an interpretation of **x**.

1 $$\xi \left( x \right) = \begin{array}{*{20}{c}} {\arg \min } \\ {g \in G} \end{array}\Gamma \left( {h,\;g} \right) + \Omega \left( g \right) = \mathop {\sum}\limits_{i = 1}^M {\left( {h\left( {x^{\left( i \right)}} \right) - {\rm{B}}x^{\left( i \right)}} \right)^2 + \mathop {\sum}\nolimits_j {\left| {\beta _j} \right|} },$$

where *h* represents the trained DNN, and *g* is the explanation model. ${\mathrm{\Gamma }}\left( {h,\,g} \right)$ represents the fidelity of the explanation model *g* and the DNN *h*. $\Omega \left( g \right)$ is the interpretability of the explanation model. The coefficient $\beta _j$ indicated the contribution of a feature to the DNN decisions around the patient **x**. Therefore, by comparing the value of the coefficients, we could have better insights on how the model decisions were made and what were the driving features of the patient. To be more specific, a positive influence meant that the feature contributed to a positive decision whereas a negative influence meant that the feature contributed to a negative decision.

## Results

### Model performance

**Fig. 3:**
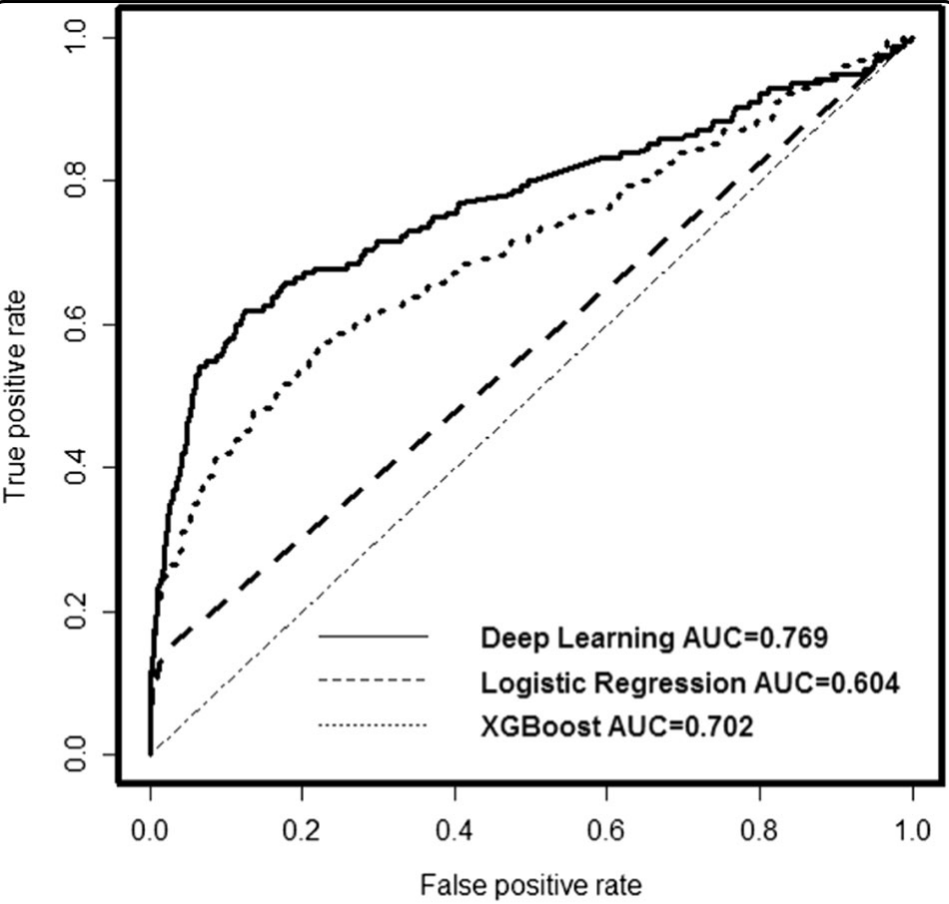
ROC curves of three different algorithms applied on the prospective cohort. The AUC of the deep learning model is 0.769, the AUC of the logistic regression model is 0.604, and the AUC of the XGBoost model is 0.702. The deep learning model has highest AUC and best performance compared to the logistic regression model and the XGBoost model.

The AUC ROC of the Deep Learning model was 0.769 (95% CI: 0.721–0.817) in the independent prospective cohort, indicating that the model was acceptable (Fig. 3). The AUC ROC curves of other models built with some popular algorithms, such as XGBoost (AUC 0.702, 95% CI: 0.652–0.751) and L-1 regularization logistic regression (AUC 0.604, 95% CI: 0.564–0.632), on the same datasets were also shown in Fig. 3, which indicated that the Deep Learning model performed better than XGBoost (*p* value 0.05) and logistic regression (*p* value < 0.0001).

Patients were stratified into four different risk groups where the stratum-specific likelihood ratio (SSLR) monotonically increased from lower than 1 to over 10, indicating patients who likely to have suicide attempts were separated from those likely to be normal (Supplementary 4). The “low” risk group, which contained the largest number of patients (*N* = 109,793), had the lowest rate of suicide attempts in next 1 year (0.11%; 119 of 109,793) and the lowest SSLR (0.630). SSLR increased dramatically in the “high” risk group and reached a peak at the “very high” risk group (9.49 and 65.57, respectively). Large SSLR values indicated patients in these two groups were more likely to have suicide attempts compared to the baseline. PPV (1.61% and 10.14% in “high” and “very high”, respectively) and relative risk (9.35 and 59.02, respectively) additionally supported such risk stratification.

Using the DNN on the EHR-based data, our prediction model found that suicide attempts patients were more likely to be in age groups of 6–54, to have diagnosed mental health conditions or pain, to have previous suicide attempts, to have been treated by psychotropic medications, and to have open wounds or injuries due to unspecific reasons. In general, a total of 117 features were significant in the predictive model, including 1 demographic feature, 1 clinical utilization measure, 73 diagnostic codes, 24 procedure codes, 7 medication prescriptions, and 11 socioeconomic characteristics (Supplementary 5). The performance of model decision interpretation is demonstrated using three representative individuals from the prospective cohort (Supplementary 6).

### Survival analyses

In this study, we adopted a 12-month time horizon to predict patients’ suicide attempts. However, it is also wondered whether our model’s performance, as well as the involved predictors, would still be the same if a much shorter time period was introduced. To uncover the impact of time horizon on the model’s performance, we explored the survival curves for the identified risk categories (high/very high, medium, and low) derived from our model, and compared their patterns within different time periods (Supplementary 7). The results showed that the three risk categories have distinct survival patterns over the 1-year time period. Even when the time horizon was cut down to 1 month and 3 months, the constructed prediction model was still able to distinguish the patients with high/very high risk of suicide attempts from those of low risk, revealing the model’s robustness regarding the outcome’s time frame adjustments. Moreover, when focusing on the real cases of suicide attempts in our prospective cohort, with a total of 16 patients that had suicide attempts within the 1-month period, 12.5% (2/16) and 25% (4/16) of them were successfully captured by our model as the high-/very-high- and medium-risk patients, respectively. When the outcome’s time period extended to 3 months, our model successfully identified 12.5% (7/56) and 14.3% (8/56) of cases as the high-/very-high- and medium-risk ones of suicide attempts, while the sensitivities increased to 18.2% (37/203) and 23.2% (43/203) for the 1-year period. However, the suicide attempt rate became low when a shorter time frame was used. Therefore, such analytics was not performed.

### Time decay analyses

Time decay is quite typical for EHR-based data, and thus, the recall period of the predictors may influence the results of prediction^22^. To reveal this, we extracted our predictors back to 3.5 years before suicide attempt and calculated the case subjects’ cumulative risk scores in months over the time spectrum. It turned out that their risk scores would become elevated about 3 years before the case event, indicating an association between the recall period of predictors and the risk scores for cases (Supplementary 8). As a limitation of the study, the development and the validation of the model were constrained by a 12-month recall period. Model performance could be influenced by time decay effect in predictors when applying it to a longer time frame out of the 12-month period.

## Discussion

Suicide attempt prediction has been challenged by low incidence for years^24^. A risk-stratification model, even though is able to identify patients with much higher risk of an event than the population mean, may still fail in producing a fairly high PPV^22^. SSLR analysis was formally introduced to evaluate the model in addition to PPV and AUC. SSLR is an approach independent of the incidence^36,37^. It describes the change from the prior probability to post-test probability within each risk category. Although the PPVs in “high” and “very high” risk categories were 1.61% and 10.14%, respectively, the SSLR values were 9.5 and 65.6 in these two categories. It meant compared with the pre-test odds, the post-test odds in “high” and “very high” risk categories have been increased by more than 9 times and more than 65 times, respectively. The SSLR approach gave a quantitative measure of the elevated risk in the “high-risk” group, and indicated that attention should be paid to the patients in that group for suicide prevention.

Our model had comparable performance to a previous EHR-based predictive model of suicide behavior in a general population^22^, in terms of the AUC (0.769 vs. 0.77) and sensitivity (41% at 93% specificity vs. 33% at 95% specificity). The PPV was lower (1% at 93% specificity vs. 6% at 95% specificity) due to the lower incidence of the cases (0.17% vs. 1.2%). However, the relative risk (the ratio of PPV to incidence) of our model was higher (5.9 vs. 5.1). Similarly, compared with other machine-learning algorithms developed and tested with case-enriched cohorts^17,19^, our model had a lower PPV but much higher relative risk (5.9 vs. 2.8^19^ and 1.3^17^).

Established suicide risk attempt factors include anxiety disorders^38,39^, bipolar disorders^40^, substance abuse^6,41,42^, pain^43^, disability^44^, bereavement^45^, and more. Prior studies have predominantly examined the clinical and statistical relationship among these risk factors and suicide attempts through univariate analysis or simple logistic regression. Epidemiological research has provided predictive analyses regarding demographic features, symptoms, and diagnoses^3,46–48^. Several authors have shown that it is possible to predict suicide risk for the patients in the Veterans Health Administration based on their EHR^3,46^.

Although EHR contains tremendous information about a patient’s clinical history, it has historically proven difficult to extract insight from the predominance of nonstructured data fields inherent to the EHR. Moreover, the connection between the clinical history and the clinical outcome is highly nonlinear and can be tangential. Traditional machine-learning algorithms either fail to capture the nonlinear relationships or lose generalizations on prospective datasets. Deep learning techniques have recently demonstrated tremendous success in many fields with its superior predictive capabilities^49–53^. Deep learning algorithms are well suited to uncover and recognize (learn) the hidden explanatory factors of variation behind complex data and simultaneously maintain the utility of generalization. By utilizing deep learning as a large-scale population screening tool, the EWS developed in this study utilizes an individual’s immediate prior 1-year clinical information, combined with available regional data related to socioeconomic determinants, to predict the suicide attempt probability within the next 12 months. The potential benefits of this approach include a reduction in manual case reviews and surveys, precise risk stratification according to absolute risk expressed as probability, alerting medical care practitioners to the need for mental health specialists for a better integrated care plan, and a method to facilitate the effectiveness of risk mitigating interventions through targeting for providers in near real time and longitudinally.

In addition, the proposed method can provide implications for treatment from two aspects. Firstly, the identified risk factors can help physicians understand the common profiles and patterns of the patients at elevated risk, so as to develop suicide prevention strategic planning and interventions. For example, providing medication and psychological treatment for the patients with mental disorders, and for those patients with disability or bereavement, the efforts and help from the community would be very helpful. Secondly, the decision interpretation work can help physicians understand the driving features of individual patient, thus design personalized intervention programs. For example, if a patient is elevated because he has committed suicide attempt before as well as he has depression disorders, involving people with lived experience in decisions about their own treatment and care with the patient would be a good complement to just medication treatment.

### Interpretation of meaningful findings and its implementations for prevention and early intervention

#### Mental illness

**Fig. 4:**
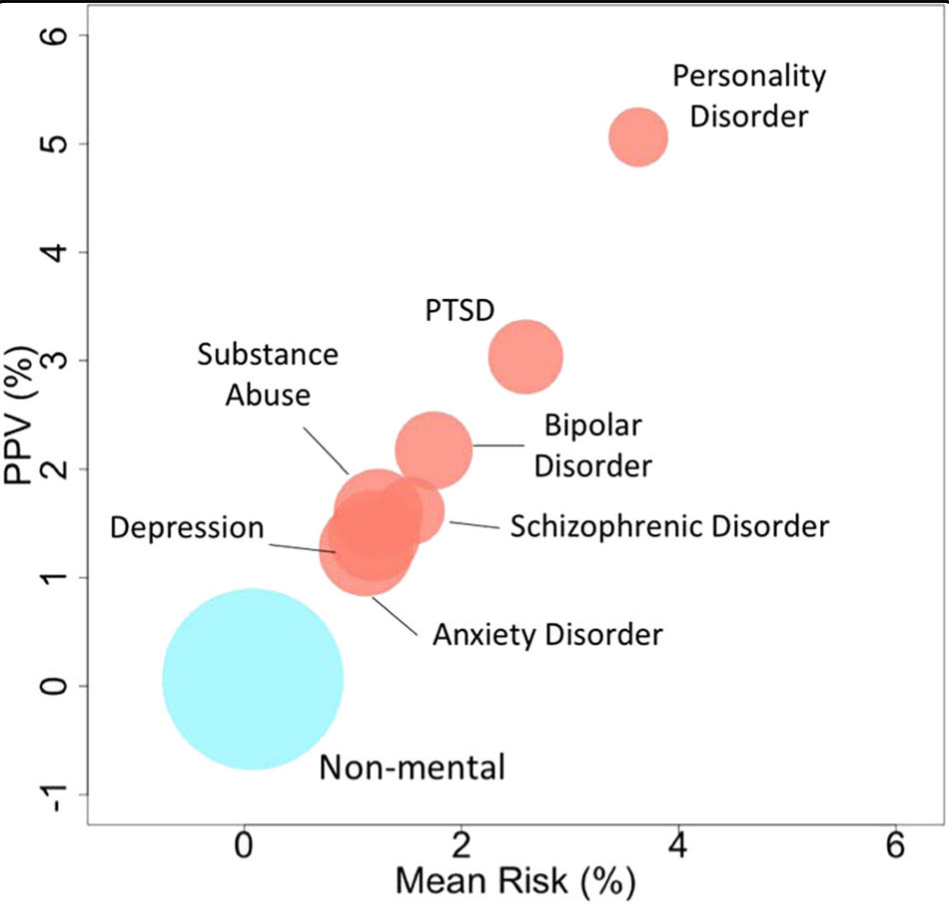
Mental illness subgroup’s average risk against the PPV. The centers of the circles were the mean risk and PPV values. The radius represented the number of individuals in each subgroup, the bigger the radius was, the more individuals the subgroup had. Each circle was also a pie chart, that represented the gender distribution in each subgroup.

Most individuals in the “very high risk” group suffered from at least one mental illness condition, and, in the literature, of those who died from suicide, more than 90% had a diagnosable mental illness^54^. The strong association between mental illness, suicide and suicide attempts has been observed in many prior studies^6,39,40,42,55^. Additionally, our model also provides strong evidence of the association between mental illness and suicide attempt behavior. The risk of suicide attempt among the people with at least one mental disorder is more than ten times greater than those without (Fig. 4).

#### Socioeconomic features

Considering socioeconomic features, in the current study, white and native American groups, with either public or private VA-related insurance, had an observed higher relative risk of suicide attempt compared to other demographics. It is well established that overall health status differs greatly depending on where people are born, live and work^56,57^. Community-level social determinant features including socioeconomic status, education level, employment status, family income, community, and environment serve as proxies and illustrate the influence between living resources, health status and health outcomes. In previous research, socioeconomic disadvantages including high poverty, high deprivation, and high unemployment were found to have a strong link to suicidal behaviors^57,58^. Consistent with previous studies, our model demonstrates that suicide attempt rate increases in communities with high unemployment and low household income.

#### Age-related feature difference

In the Berkshire datasets, most suicide attempts occur in the age group of <25 and the age group of 25–54 (Supplementary 9). This observation is consistent with the recently published study out of Spain^11^. Global and national trends and the Parra-Uribe study indicate that suicide rates increase with age and peak in older adults^59^. Using our deep learning methodology with a dataset that includes actual suicides in the Berkshire population may provide important insights into the difference between risk factors for suicide attempt and risk factors for completed suicide.

**Fig. 5:**
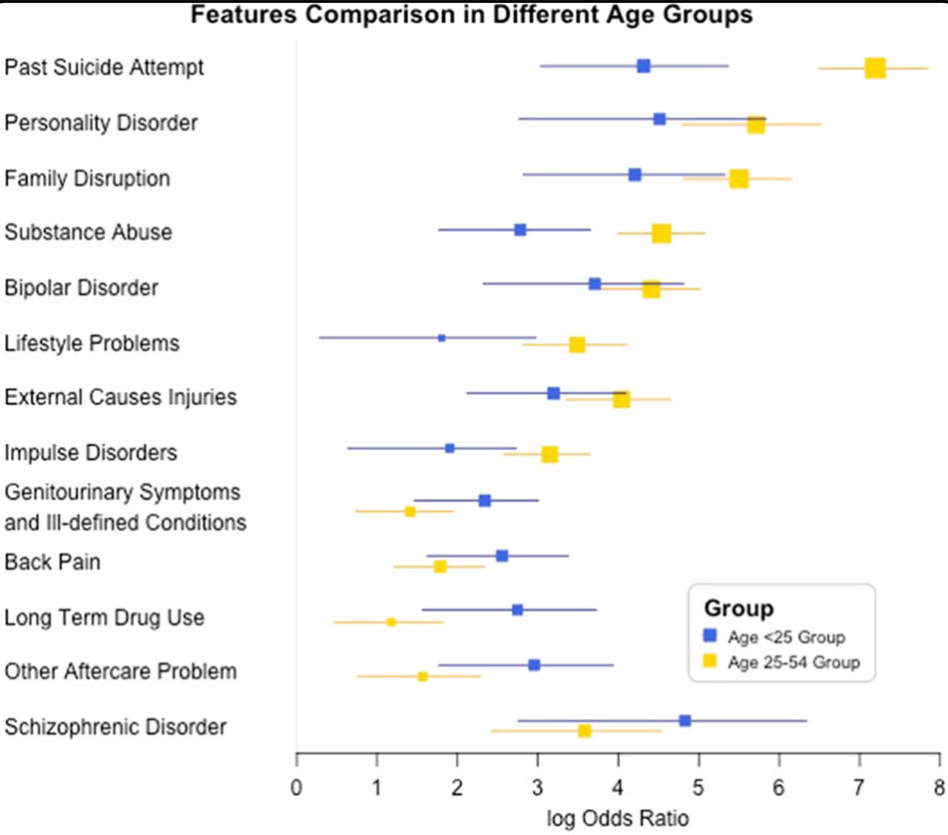
Forest plot of odds ratios (and their 95% confidence intervals and *p* value < 0.01, the size of the square is proportional to the negative log *p* value) for the comparison between the <25-year-old age group and the 25–54-year-old age group. Past suicide attempt is the strongest risk factor for the age group of 25–54, while mental disorders like personality disorder, substance abuse, and bipolar disorder also have more influence on this age group. In the younger group, the physiological defects and the pain-related disorders are stronger predictors.

It is found that different features influence the risk differently in these two age groups (Fig. 5), suggesting that the suicide prevention/intervention should have different strategies or focus. Past suicide attempt is the strongest risk factor for the age group of 25–54, while mental disorders like personality disorder, substance abuse, and bipolar disorder also have more influence on this age group. Therefore, the suicide prevention strategies of the 25–54 age group should focus on the diagnosis and care of conditions. In the younger group, the physiological defects and the pain-related disorders are stronger predictors, which suggests that medical care to manage pain and other conditions is as important as identifying and managing mental health disorders. It may also be that the presence of an unexpected medical condition or pain syndrome in a young person is more difficult to accept or live with than it is in an older person who may have more peers with such afflictions and who anticipates that these events come with older age. Thus, psychotherapeutic attention to the meaning of the unexpected and, perhaps isolating, medical condition for the young person may be an important point of intervention.

#### Decision interpretations

Most of the false positives possess many strong risk predictors, indicating the individuals share characteristics with individuals who do attempt suicide. These patients should have outreach interventions to mitigate suicide attempt risk in subsequent years. The Bostwick report showed that 73% of those who died on their index attempt used firearms, and the odds ratio for gunshot vs. all other methods was 140^9^. Lethal means restriction would be an important part of outreach and support to those identified at high risk of a future attempt^16^. Our model aims to predict suicide attempt in the next 1 year, but mental disorders and suicidal behavior may develop as an accumulating burden over a more extended period of time.

#### Study limitations

There are several limitations of this study. The first was the case definition. Uncoded suicide attempt cases could be outliers of the model and affect accuracy. The uncoded suicide attempts come from two parts. One is those who died on their first attempt, whose data would not appear in the EHR database. The other is the unreported suicide attempts. But the Berkshire Health System is taking some actions to make the situation better^60^, like training the hospital staff to look for warning signs of suicide attempt and ask follow-up questions to see if the patient might be an unreported suicide attempt.

The second limitation is the misclassifications of the model. The PPV of the model was constrained by the low incidence of the suicide attempts; however, the SSLR analysis and results supported the risk stratification. As stated above, the false negatives are mainly due to the missing data issue in the EHR dataset. This can be solved by involving other dataset. As for the false positives, we noticed that most (over 50%) of the false positives had substance abuse (73.1%), lifestyle problems (67.3%), history of mental health and substance abuse (57.1%), depressive disorders (54.6%), and personality disorders (51.9%), and we also did a *t* test between the features in the high-risk false positives and the true positives. The large *p* values, i.e. substance abuse (0.85), lifestyle problems (0.35), history of mental health and substance abuse (0.55), depressive disorders (0.36), and personality disorders (0.56) show that there is no true difference of the features between the false positives and the true positives. Therefore, most of the false positives possess many strong risk predictors, indicating the individuals share characteristics with individuals who do attempt suicide. These patients should have outreach interventions to mitigate suicide attempt risk in subsequent years. The Bostwick report showed that 73% of those who died on their index attempt used firearms, and the odds ratio for gunshot vs. all other methods was 140^9^. Lethal means restriction would be an important part of outreach and support to those identified at high risk of a future attempt^16^. Our model aims to predict suicide attempt in the next 1 year, but mental disorders and suicidal behavior may develop as an accumulating burden over a more extended period of time.

Third, the study did not examine mortality associated with subsequent suicide attempts. There were a number of patients who attempted suicide, who were treated either in the Emergency Department (ED) or ED and acute hospital, who died in the ED or acute hospital after their attempt. The number was too small for our model to be able to detect a pattern. Future studies with a larger sample of suicide attempts and a greater number of actual suicides would be valuable in addressing the important question of differences among those who attempt suicide and die on the first attempt, those who die on a subsequent attempt, and those who survive multiple attempts.

## Conclusions

In summary, incorporating EHR-based suicide attempt risk models in routine medical care practice will have no extra cost on data collection and can be applied in a straightforward manner. The proposed model can be used at every step in preventing suicide attempts and hopefully, in also preventing suicide. This approach can identify high-risk individuals from the EHR in population scale, from which care managers and clinicians can develop personalized intervention plans by interpreting the predictive decision, learning about the driving risk factors, tracing the progress of the intervention process, and through this process, decrease suicide attempts and likely save lives.

## Supplementary information

Supplementary

## Data Availability

Code used for the analyses described in this manuscript is available from the corresponding author upon request.
