# Supplementary material for: Development of an early-warning system for high-risk patients for suicide attempt using deep learning and electronic health records
Source: Transl Psychiatry. 2020 Feb 20;10:72. doi: 10.1038/s41398-020-0684-2 (PMC7033212; doi:10.1038/s41398-020-0684-2)
Supplement: Supplementary file 1 — Supplementary [file 41398_2020_684_MOESM1_ESM.docx]

#### Supplementary 1: Suicide attempt case distribution in different mental illness


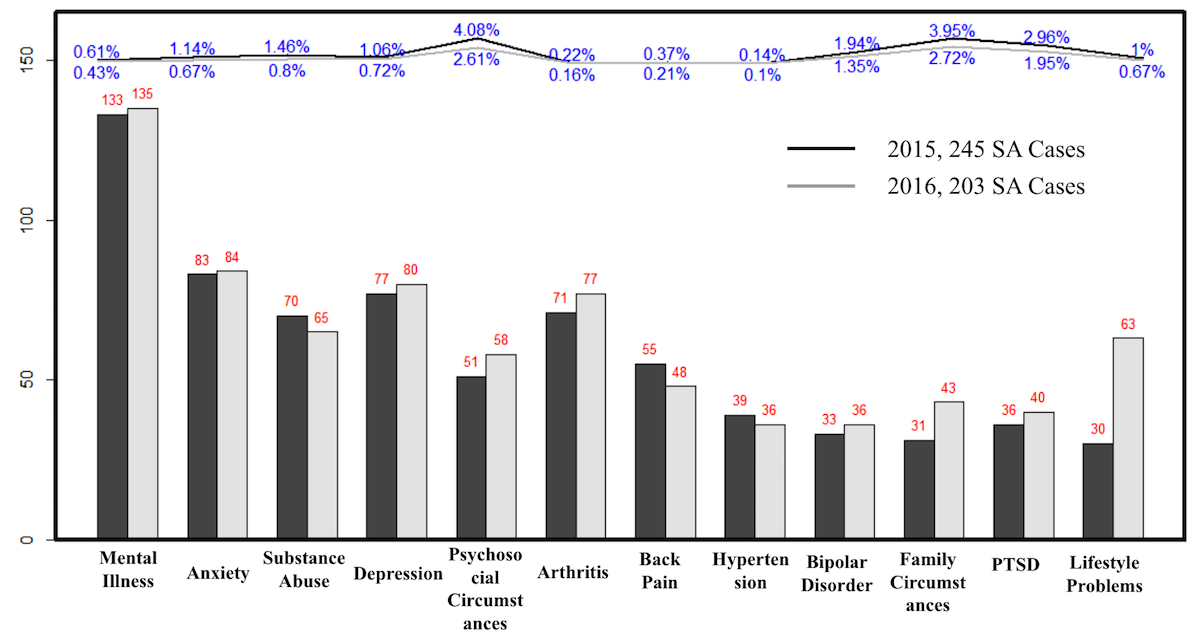


Supplementary Figure 1. Sub-cohort enrichment of incidence rate with different disease burden in 2015 and 2016, respectively

#### Supplementary 2: Baseline characteristics of the training and testing cohorts

Supplementary Table 1 Baseline characteristics of the cohorts

| **Characteristic** | **Retrospective cohort** | **Training**  **cohort** | **Validation**  **cohort** |
| --- | --- | --- | --- |
| **n(%)** | **(N=118,252)** | **(N=21,013)** | **(N=118,095)** |
| Age |  |  |  |
| <=18 | 16,960 (14.34%) | 1,424 (6.78%) | 16,614 (14.07%) |
| 19-40 | 28,104 (23.76%) | 6,177 (29.40%) | 28,121 (23.81%) |
| 41-65 | 44,860 (37.94%) | 8,255 (39.28%) | 44,524 (37.70%) |
| >=66 | 28,328 (23.96%) | 5,157 (24.54%) | 28,836 (24.42%) |
| Gender |  |  |  |
| Male | 53,416 (45.17%) | 9,781 (46.55%) | 53,287 (45.12%) |
| Female | 64,836 (54.83%) | 11,232 (53.45%) | 64,808 (54.88%) |
| Suicide attempt | 245 (0.21%) | 133 (0.61%) | 233 (0.20%) |

#### Supplementary 3: Missing data handling

Before constructing the deep learning model, all the missing data were preprocessed. Missing data was a data integrity problem that existed widely throughout the EHR. For example, there were 3.7% of encounters with missing geographic information, 0.56% with missing gender or age, and 0.33% with missing admission or discharge date. In deep learning techniques, deep denoising autoencoder is designed to recover clean output from noisy input [1]. Since missing data is a special kind of noisy input, we used a four-layer deep denoising autoencoder to impute the missing demographic data [2, 3]. There were also possibly some longitudinal data of medical histories missing. It is a study limitation. However, from a deep learning point of view, if we assumed that the missing medical histories were totally random and replaced them with zeros, they behaved in a way similar to dropout mechanism, which could provide regularization to the deep neural network and avoid overfitting [4].

#### Supplementary 4: Risk stratification by the SSLR method

Supplementary Table 2. Model performance by risk groups in the prospective cohort. SSLR: stratum-specific likelihood-ratio.

| **Risk Group** | **Abnormal** | **Normal** | **SSLR**  **(95% CI)** | **PPV%** | **Relative Risk** |
| --- | --- | --- | --- | --- | --- |
| Low | 119 | 109,674 | 0.63  (0.56-0.71) | 0.11 | 0.63 |
| Medium | 47 | 6,686 | 4.08  (3.17-5.25) | 0.70 | 4.06 |
| High | 23 | 1,408 | 9.49  (6.43-13.99) | 1.61 | 9.35 |
| Very High | 14 | 124 | 65.57  (38.39-111.98) | 10.14 | 59.02 |

#### Supplementary 5: Categories of significant features


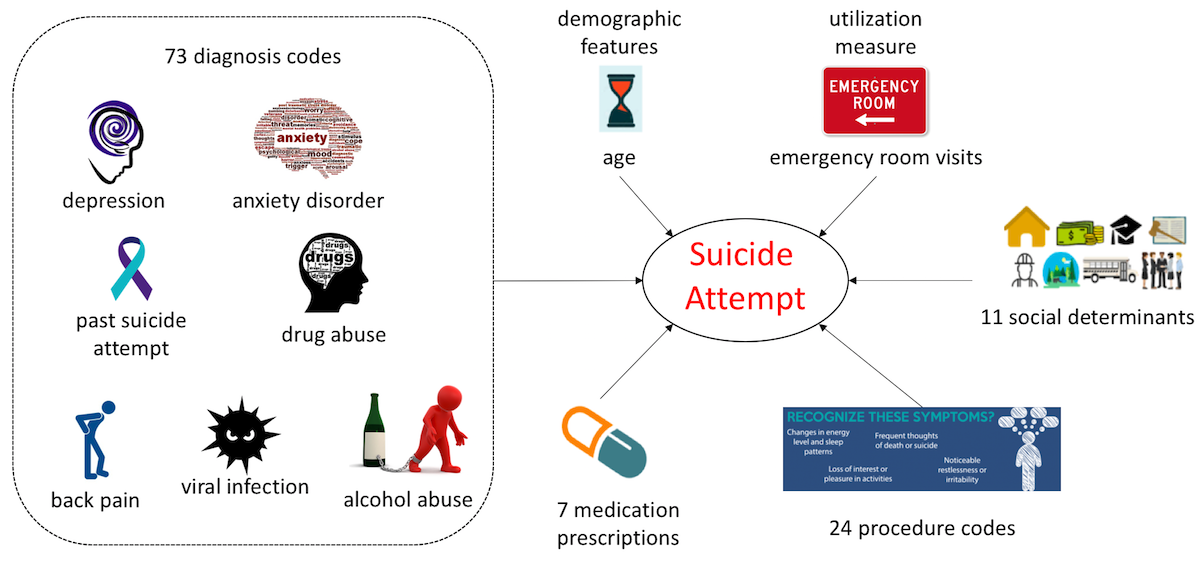


Supplementary Figure 2. Six categories of the 117 EHR-based significant features used in the risk model.

#### Supplementary 6: Examples of model interpretation

We demonstrated the decision interpretations for three representative individuals from the prospective cohort, i.e. a true positive (Supplementary Table 3), a false positive (Supplementary Table 4), and a false negative (Supplementary Table 5), to illustrate the effect of the interpretation method. The influences were the coefficients computed from equation (1) in the main text. And features =1 denoted the individual had the feature in the past one year whereas features =0 indicated that the individual did not have the feature. The main findings are:

1. For the true positive, the interpretations indicated that making a suicide attempt, suffering from borderline personality disorder, and prescribed a bronchodilator were the driving features to make the prediction. Bronchodilator is a medication to treat or prevent bronchospasm, which often affects people with asthma and allergies, and contributes to asthma symptoms like wheezing and shortness of breath. Thus, the interpretation indicated that asthma could also increase the risk of suicide, which was consistent with previous studies [5].
2. For the false positive, the driving features were past suicide attempt in past 1 year and 3 years, suicidal ideation, and symptoms involving digestive systems. Though this patient did not commit suicide in the next one year, attention should be paid to this patient and certain interventions should be applied to mitigate the event in the following years.
3. For the false negative, the interpretations showed that the patient was predicted as low risk because the patient did not have several key features of suicide attempt. In fact, over 20% (48/203) of the true cases had no admission until they were admitted due to the suicide attempt event. This was a limitation of our study since those patients were all assigned to the low risk group. A broader range of data, like the social media data [6], could be included and help build a better prediction model.

Supplementary Table 3 Decision interpretations of a true positive with risk score 0.89

|  | Influence | Description |
| --- | --- | --- |
| Positive | 0.11 | Diagnosis: Suicide and self-inflicted injury by cutting and piercing instrument |
|  | 0.11 | Diagnosis: Open wound of wrist |
|  | 0.10 | Medication: Treat or prevent bronchospasm |
|  | 0.07 | Diagnosis: Borderline personality disorder |
|  | 0.06 | Diagnosis: Other specified family circumstances |
| Negative | -0.03 | Diagnosis: Open wound of forearm |

Supplementary Table 4 Decision interpretations of a false positive with risk score 0.65

|  | Influence | Description |
| --- | --- | --- |
| Positive | 0.08 | Diagnosis: Open wound of forearm |
|  | 0.07 | Diagnosis: Suicide and self-inflicted injury by cutting and piercing instrument |
|  | 0.06 | Diagnosis: Suicide ideation |
|  | 0.06 | Diagnosis history: Past suicide attempt in past 3 years |
|  | 0.03 | Diagnosis: Nausea |
| Negative | -0.05 | Diagnosis: Episodic mood disorder |

Supplementary Table 5 Decision interpretations of a false negative with risk score 0.02

|  | Influence | Description |
| --- | --- | --- |
| Positive | 0.01 | Diagnosis: Suicide ideation |
| Negative | -0.03 | Utilization: Emergency room visit > 5 times in past one year |
|  | -0.04 | Diagnosis: Alcohol-induced mental disorders |
|  | -0.04 | Diagnosis: Episodic mood disorder |
|  | -0.05 | Diagnosis: Acute alcoholic intoxication in alcoholism |
|  | -0.06 | Diagnosis: Depressive disorder |

#### Supplementary 7: Survival analyses


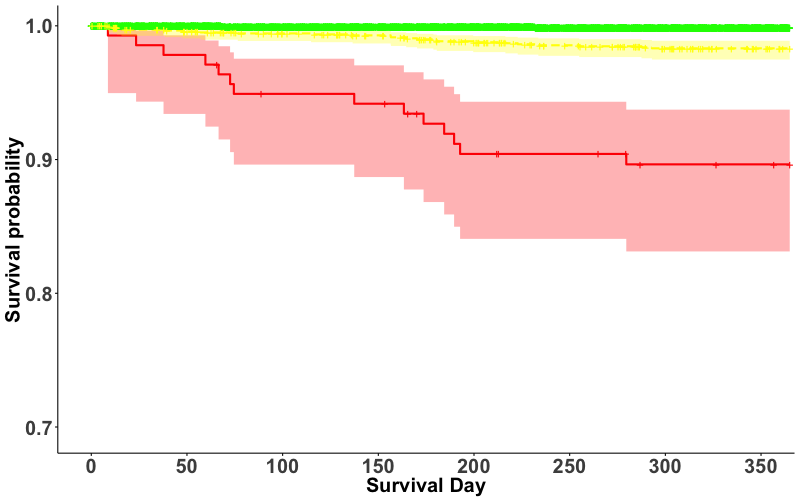


Supplementary Figure 3. Survival curves of the low (green)-, medium (yellow)-, and high/very high (red)-risk groups in the prospective cohort. The differences between the three risk groups within a 1-month, 3-month, and 1-year period are all significant (P < 0.001).

#### Supplementary 8: Time decay analyses


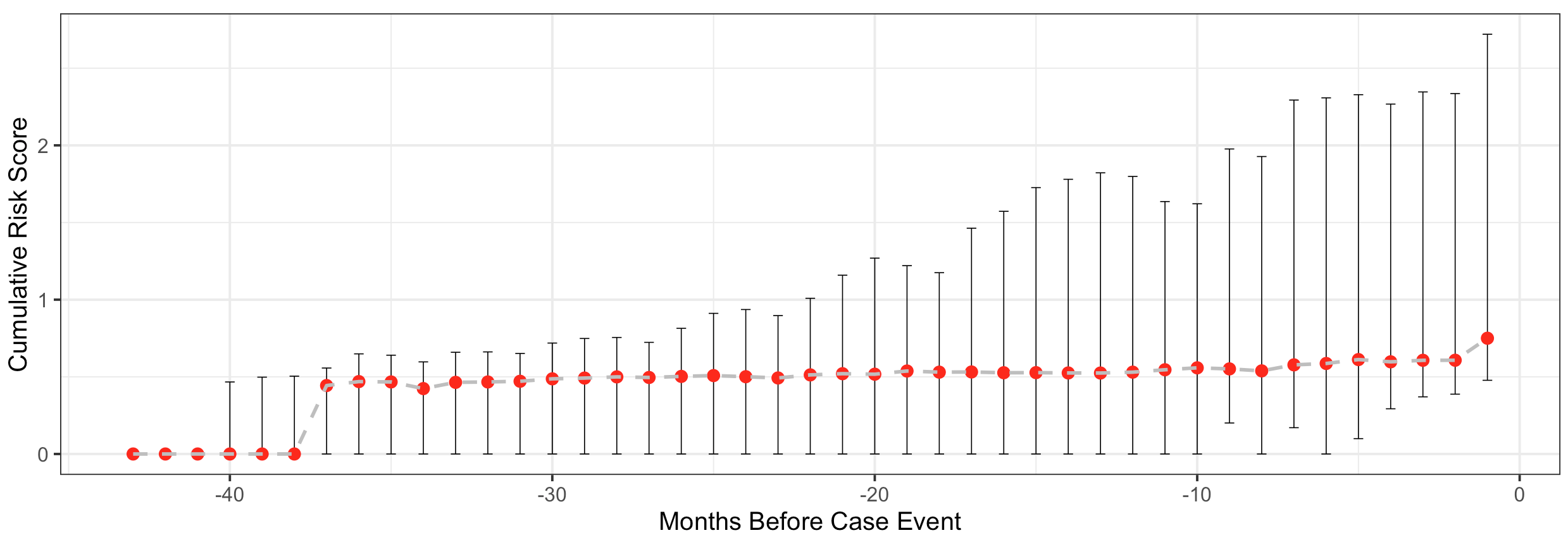


Supplementary Figure 4. Cumulative risk scores of prospective patients with suicide attempts as a function of months to the attempts occurred.

#### Supplementary 9: Risk distribution in different age groups


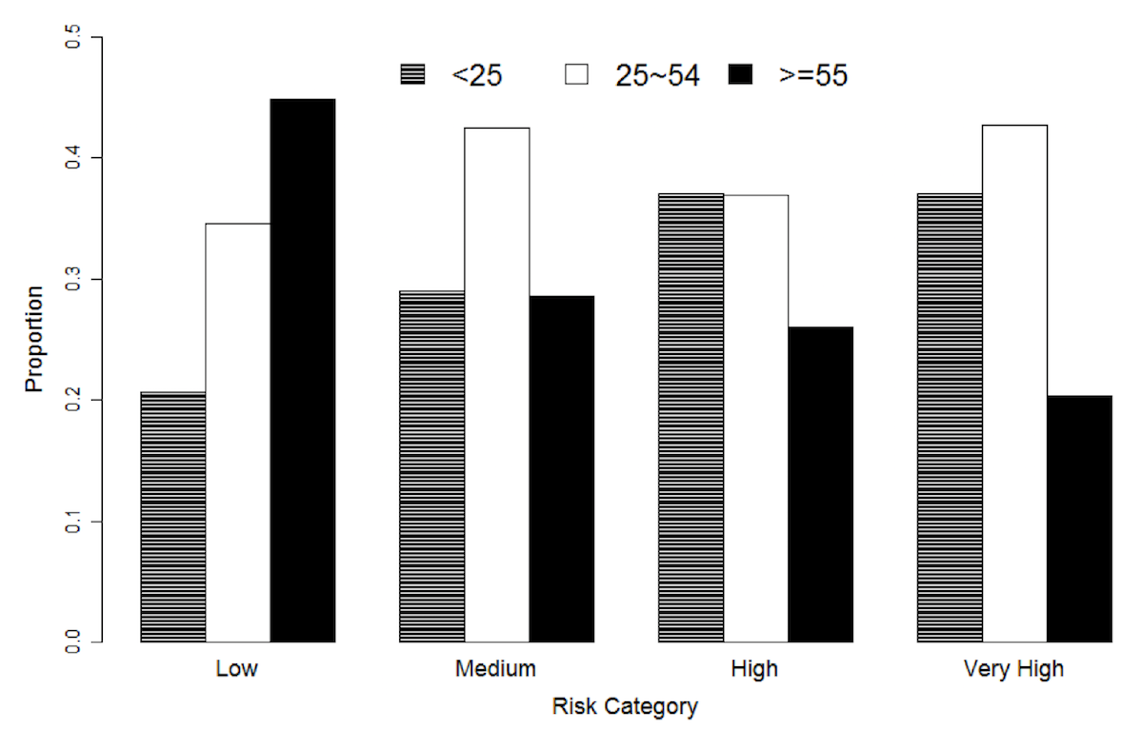


Supplementary Figure 5. Constituent ratios of age subgroups across the identified four risk categories. Age groups: <25, 25-54, and ≥55

#### Supplementary 10: Model Interpretation via Mimic Model Method

In addition to FDR and odds ratios, we also evaluate the importance of the features using a decision tree model (XGBoost). XGBoost identified past suicide attempt, substance abuse, arthritis excluding back pain, anxiety disorder and back pain as the top-5 strongest predictors of suicide attempt.

Supplementary Table 6 Feature importance values calculated by XGBoost.

| **Feature Description** | **Feature Importance** |
| --- | --- |
| Past suicide attempt | 0.16917 |
| Substance abuse | 0.11553 |
| Arthritis excluding back pain | 0.047 |
| Anxiety disorder | 0.03274 |
| Back pain | 0.03258 |
| Vehicle accidents at home | 0.02932 |
| Headache | 0.02785 |
| Female | 0.02401 |
| PTSD | 0.02205 |
| Unspecified injuries | 0.0215 |
| Other ill-defined and unknown causes of impulsiveness | 0.02067 |
| Esophageal disorders | 0.02056 |
| Depressive disorder | 0.0198 |
| Essential hypertension | 0.0179 |
| Screening and history of mental health and substance abuse codes | 0.01641 |
| Unspecified injuries from unspecified external causes | 0.01616 |
| Unspecified episodic mood disorder | 0.01616 |
| Personal history of noncompliance with medical treatment, presenting hazards to health | 0.01592 |
| Family history of psychiatric condition | 0.01518 |
| Mood disorders | 0.0149 |
| Vehicle accidents at unspecified place | 0.013 |
| Esophageal reflux | 0.01295 |
| Serotonin Reuptake Inhibitor | 0.01292 |
| Health influenced by physical restraints | 0.01228 |
| Anxiety disorders, unspecified | 0.01156 |
| Acute alcoholic intoxication in alcoholism | 0.01109 |
| Alcohol-related disorders | 0.01104 |
| Family problems | 0.0106 |
| Major depressive affective disorder, recurrent episode | 0.00991 |
| Educational circumstances problems | 0.00985 |
| Injury and poisoning activities involving snow and ice | 0.00968 |
| Open wound of wrist | 0.00968 |
| Long term drug use | 0.00922 |
| Knee, leg, ankle, and foot injury | 0.0087 |
| Psychosocial issues | 0.00844 |
| Vehicle accidents, unspecified | 0.00832 |
| Depression, unspecified | 0.00775 |
| Lifestyle problems | 0.007 |
| Anxiety state | 0.00688 |
| Suicide and self-inflicted poisoning by tranquilizers and other psychotropic agents | 0.00622 |
| Pain | 0.00611 |
| Open wound of forearm | 0.00606 |
| Unspecified injuries from other external causes | 0.006 |
| Asthma | 0.00526 |
| Schizophrenia and other psychotic disorders | 0.00523 |
| Unspecified essential hypertension | 0.00522 |
| Joint pain | 0.00477 |
| Tobacco use disorder | 0.00475 |
| Other nervous system disorders | 0.00454 |
| Personal history of other injury | 0.0045 |
| Unemployment | 0.00447 |
| Suicidal ideation | 0.0044 |
| Neuropathy | 0.00423 |
| Generalized anxiety disorder | 0.00409 |
| Bipolar disorder | 0.00394 |
| Developmental disorders | 0.00292 |
| Fibromyalgia | 0.0027 |
| Suicide and self-inflicted injury by cutting and piercing instrument | 0.00259 |
| Impulse control disorders | 0.0021 |
| Bipolar disorder | 0.00209 |
| Suicide and self-inflicted poisoning by other specified drugs and Medicinal substances | 0.00183 |
| Other alcohol-induced mental disorders | 0.00155 |
| Personality disorders | 0.00137 |
| Other endocrine disorders | 0.00122 |
| Pain in joint, forearm | 0.00103 |
| Migraines | 0.00056 |
| Borderline personality disorder | 0.00001 |

#### Supplementary 11: Modeling in different age groups

Supplementary Table 7 displays the model performance in two age groups (<25 and 25-54) of the prospective cohort. The model has a higher sensitivity (21.7% vs 16.1%; p-value = 0.4) but a lower PPV (2.7% vs 4.8%; p-value = 0.1) with patients <25. Such differences, however, are not significant. We also explored our modeling in different age groups (Supplementary Table 8). Compared with our population model, the specific models developed within each age group demonstrated a better PPV with patients at 25-54 (3.3% vs 2.7%; p-value = 0.5), but worse sensitivity and PPV with patients <25 (sensitivity: 13.6% vs 16.1%; P = 0.8; PPV: 2.7% vs 4.8%; p-value = 0.2). The reduced performance of the model developed with patients <25 is probably due to the small number of cases (n=13).

Supplementary Table 7 The performance of a single model in different age groups in the prospective cohort

|  | Sensitivity | Specificity | PPV | NPV |
| --- | --- | --- | --- | --- |
| Age Group <25 | 16.1% | 98.7% | 4.8% | 99.7% |
| Age Group 25-54 | 21.7% | 98.1% | 2.7% | 99.8% |
| Age Group >54 | 12.5% | 99.1% | 0.6% | 99.9% |

Supplementary Table 8 The performance of models developed within different age groups separately

|  | Sensitivity | Specificity | PPV | NPV |
| --- | --- | --- | --- | --- |
| Age Group <25 | 13.6% | 98.0% | 2.7% | 99.6% |
| Age Group 25-54 | 20.6% | 98.5% | 3.3% | 99.8% |

#### Reference

1. Vincent, P., et al. *Extracting and composing robust features with denoising autoencoders*. in *25th international conference on Machine learning*. 2008.

2. Beaulieu-Jones, B.K. and J.H. Moore, *MISSING DATA IMPUTATION IN THE ELECTRONIC HEALTH RECORD USING DEEPLY LEARNED AUTOENCODERS.* Pac Symp Biocomput, 2017. **22**: p. 207-218.

3. Gondara, L. and K. Wang, *MIDA: Multiple Imputation using Denoising*

*Autoencoders*. 2017.

4. Srivastava, N., et al., *Dropout: A Simple Way to Prevent Neural Networks from*

*Overfitting.* Journal of Machine Learning Research, 2014. **15**: p. 1929-1958.

5. Chung, J.H., S.H. Kim, and Y.W. Lee, *Suicidal ideation and suicide attempts among asthma.* Ann Gen Psychiatry, 2016. **15**: p. 35.

6. Choudhury, M., et al., *Predicting Depression via Social Media*, in *The Seventh International AAAI Conference on Weblogs and Social Media*. 2013: Cambridge, Massachusetts.
